# How Batter Formulation Can Modify Fried Tempura-Battered Zucchini Chemical and Sensory Characteristics?

**DOI:** 10.3390/foods9050626

**Published:** 2020-05-13

**Authors:** Montserrat Martínez-Pineda, Cristina Yagüe-Ruiz, Antonio Vercet

**Affiliations:** Faculty of Health and Sports Science, University of Zaragoza, Plaza Universidad, 3. 22002 Huesca, Spain; cyague@unizar.es (C.Y.-R.); vercet@unizar.es (A.V.)

**Keywords:** coating, ethanol, hydrocolloid, maltodextrin, oil, crispiness, tempura-batter, organoleptic characteristics

## Abstract

Tempura-fried vegetables are widely consumed and are greatly appreciated because of their characteristic dry and crispy crust, flavor and a golden–brown color. This study examined the effect of slice thickness, frying time and partial ingredient substitution in tempura batter with maltodextrin, ethanol, baking powder and cornflour on the rheological characteristics, moisture, oil uptake, color, texture and sensory characteristics of tempura-fried zucchini. The results showed an improved golden–brown coloring of the crust without affecting oil uptake when maltodextrin was included in the batter formulation. Moreover, dough viscosity and % pick-up lowered with maltodextrin addition. The partial substitution of water and wheat flour with ethanol, cornflour and baking powder resulted in a crispier and rougher crust that remained more stable over time but with less moisture and higher oil uptake. The substitution of certain tempura batter ingredients depending on the desired purpose could represent an interesting strategy to improve the quality of battered fried vegetables.

## 1. Introduction

Despite variations in culinary traditions around the world [1] fried products are appreciated worldwide, in part due to their palatability. Deep fat frying is a multifunctional process of food transformation consisting in cooking food by immersion in edible oil or fat at a temperature above the boiling point of water [2]. There is a simultaneous process of heat and mass transfer whereby water and other soluble materials are transferred from the material being fried as oil penetrates the product. This results in the typical and sought-after texture of deep-fried foods: a dry and crispy crust, with tender contents. Maillard reactions also occur in the crust, providing the flavor and color typical of fried foods [3,4].

Many fried products contain a batter coating that acts as a barrier against loss of moisture and enhances the expected organoleptic food characteristics even after freezing and reheating. A batter can be defined as a liquid dough, into which a product is dipped before it is cooked, usually by frying. A tempura-batter basically consists of a mixture of flour and water, but other ingredients are frequently added to improve texture and flavor: starches, egg, various proteins such as gluten, seasonings, gums or other hydrocolloids, or leavening agents that expand when fried [1,4].

However, fried foods contain significant amounts of fats, which in some cases account for 1/3 of the total weight of the food product [3]. From a nutritional point of view, this is a major drawback. Thus, in recent years, many innovative solutions to decrease the fat content of fried foodstuff have been proposed [5,6].

Several studies have investigated the use of hydrocolloids as thin edible coatings or as ingredients in batter formulations [1,5,7,8]. Starchy and modified starchy coatings have been proposed as an alternative to reduce oil uptake in fried foods [5,9,10]. However, no study has specifically analyzed the effect of maltodextrin addition in coating batters on oil uptake. Maltodextrins are the products of enzymatic hydrolysis of starches of various botanical origins, and they are characterized by a dextrose equivalent (DE) value of less than 20. They have a desirable filling, structuring, and stabilizing properties and also contain substances that contribute to the taste of the final food [11,12].

The use of ethanol as a batter ingredient has been proposed as a means to increase and maintain crispiness in fried products [13,14,15]. However, the few published studies on this topic focused on deep-fried battered seafoods, with no discussion of the effects on fried tempura-coated vegetables.

The objective of this study was to analyze how partial substitution of tempura batter ingredients with maltodextrin, ethanol, baking powder as a leavening agent and cornflour affected oil uptake and the sensory and physical characteristics of tempura-fried zucchini. 

## 2. Materials and Methods

### 2.1. Materials

This study was carried out using fresh zucchini (Cucurbita pepo) (52.5±2.5 mm in diameter), wheat flour (Harinera del Mar, Almenara, Castellón, Spain), cornflour (Maizena, Unilever, Viladecans, Spain), mineral water (Montepinos, Carbónicas Navalpotro, S.A., Almazán, Soria, Spain), baking powder (Hacendado, Jesús Navarro S.S., Alicante, Spain), salt and sugar purchased from a local market, as well as maltodextrin with a DE rating of 12 (Comercial Artesana Sosa Ingredients, S.L, Moià, Spain) and food-grade ethanol (Panreac Química S.L.U, Barcelona, Spain). High-oleic sunflower oil (Titan, Koipe, S.A., Gipuzkoa, Spain) purchased from a local supermarket was used for frying.

### 2.2. Batter Preparation and Assay Conditions 

The effect of slice thickness and frying time were studied. The effect of slice thickness was analyzed by slicing zucchini to a thickness of 2.25 ± 0.25 mm, 3.0 ± 0.25 mm or 4.25 ± 0.25 mm using a slicing machine (CF4821, UFESA, Barcelona, Spain); the battered zucchini were then coated with a standard tempura batter (SB), described in Table 1, and fried for 260 s at 170 °C. Slices thickness studied were frequently used in many dishes of the Mediterranean cuisine, including tempura-battered frying. To analyze frying time, 4.25 ± 0.25 mm thick and coated with SB zucchini slices were used; the battered zucchini were fried for 180, 260 or 360 s at 170 ° C.

Moreover, the effect of three different batters was analyzed: a standard batter (SB), a maltodextrin batter (MB) and a batter containing ethanol and baking powder (EB). Batter ingredients and formulations are described in Table 1. In EB wheat flour was partially replaced by cornflour to achieve enough butter consistency to cover the sample. Ingredients were combined with water at 15 °C and mixed with a kitchen mixer (Moulinex, Groupe Seb Ibérica, Barcelona, Spain) at medium speed for 45 s. For all three formulations, zucchini slices were dipped into the batter for 10 s and allowed to drip for 8 s before frying.

Differences between the three coating batters were analyzed using zucchini slices that were 4.25 ± 0.25 mm thick and deep-fried for 260 s at 170 °C.

In all cases, a deep-fryer (Magefesa, Rhointer España, Santander, Spain) with 3 L of oil was used, and only two coated slices were fried at the same time. As fried foods often exhibit altered physicochemical values when they are fried under suboptimal conditions, such as when the oil is highly degraded [16], the physicochemical characteristics (polar compounds) of the frying oil were analyzed after each assay and the oil was changed when necessary to avoid interference due to degradation compounds. The oil was discarded before the level of polar compounds reached 15%. It was measured using a polar compound tester (Testo 265, Instrumentos Testo, S.A., Barcelona, Spain).

Each assay was performed in triplicate and the number of measurements in each replication varied according to the parameter analyzed.

### 2.3. Batter Rheology Characterization

Before calculating the batter pick-up, the flow behavior of each batter preparation was analyzed. Flow behavior was analyzed immediately after batter preparation using a cone and plate rotational viscometer (Brookfield’s CAP-2000 +) equipped with a Peltier thermoelectric controller and the software CAP-CALC/CAP266Y (v. 3). We used a linear ramp varying from 0 to 267/s for the SB and MB samples, and from 0 to 330/s for the EB samples; assays were performed at 15 °C. The shear stress/shear rate data were plotted as flow curves and fitted to the Herschel–Bulkley model. The apparent viscosity, shear stress and shear rate were calculated. Eight experimental runs were performed for each assay replication (*n* = 24). 

### 2.4. Batter Pick-up Measurement

Batter pick-up denotes the amount of batter adhered to the piece of food and it is a conditioning factor in the final product characteristics. Batter pick-up was calculated as:

Batter pick-up=[(CW − IW)/IW] × 100,

where *CW* is the weight of the coated zucchini slice after dipping and *IW* is the initial weight of the uncoated zucchini slice. Batter pick-up was measured in ten slices for each assay replication (n = 30). The samples were discarded after the measurement of batter pick-up was completed. 

### 2.5. Color Determination

Color measurement was carried out using a digital image analysis system. A digital camera (PANASONIC LUMIX DMC-FZ7) was mounted on a vertical stand to provide stable support. The camera was positioned vertically over a matte black background at a distance of 32 cm. The lighting system consisted of a cold light unit (Kaiser Fototechnik, Germany) with two 36-W daylight florescent lamps with a color temperature of 5400K (Type E energy uniform illuminant) and color rendition index of 90–100. Lamps were set at approximately 45-degree angles [17]. To calibrate the digital color system, standard AENOR UNE color charts were measured. A digital image of each chart was taken in a TIFF format. RGB values for each standard were obtained with Adobe Photoshop CS3. To carry out the transformation from RGB values to the L*a*b* color space, a mathematical model was developed using Matrox 8.0 Software (Matrox Electronic Systems Ltd, Canada). Coordinate a* represents the red–green visual opponent spectrum and b* represents the yellow–blue spectrum. Coordinate L* (lightness) reflects the physiological attributes of visual response. Color parameters were analyzed in four samples for each assay replication (*n* = 12). 

### 2.6. Instrumental Texture Analysis

The determination of the textural properties of the product after frying was performed using a texture analyzer TA-XT2i (Stable Micro Systems, Goaldming, England) equipped with a 5 kg load cell. For this purpose, a Warner–Bratzler shear blade probe with a blade holder was used for the cutting/shearing test. The following settings were used for the analysis: test speed 1 mm/s, trigger force 0.005 kg and a travel distance of 10 mm. To characterize the batter layer, the following parameters were calculated from the plot of force versus time: maximum peak force (Kg), number of peaks in the initial slope or fracture events, and workforce or area under the first curve (Kg·s). 

Samples for the texture analysis were prepared using the same batter formulations and method as described above. The thickness (4.25 ± 0.25 mm) and frying conditions (260 s at 170 °C) of the samples were kept constant.

Changes in texture properties over time were also analyzed. For this purpose, texture analysis was performed both immediately and 30 min after sample preparation. Textural properties were analyzed in five samples for each assay replication (*n* = 15), and new samples were used for each time point.

### 2.7. Moisture Content 

To determine the moisture content, 15-g samples were weighed in a glass evaporating basin and dried in a laboratory drying oven (BINDER ED53, BINDER GmbH, Germany) at 105 °C for 24 h until the weight remained constant. The moisture content was calculated from the difference in weight before and after oven drying [18]. The results are expressed as percentages. These samples were then used to determine the oil content. 

### 2.8. Oil Content

To determine the oil content, dried and crushed samples were subjected to petroleum ether (Panreac Química S.L.U, Barcelona, Spain) extraction using SOXTEC equipment (FOSS Soxtec 2055, Sweden). The fat content was determined by the difference in weight before and after extraction [18]. The results were expressed as percentages.

In each assay replication, moisture and fat content were analyzed in three samples (*n* = 9).

### 2.9. Sensory Analysis

The sensory characteristics of different batters were assessed by ten experienced panelists (3 males and 7 females; 25–45 years of age) who scored the intensity of each attribute three times for each type of sample (*n* = 30).

Twelve different attributes were evaluated in the following order: external attributes (color, homogeneity of the color, roughness and homogeneity of the batter layer), general attributes (integrity and batter layer/product ratio), texture attributes (hardness, crispiness and juiciness), and flavor attributes (intensity of characteristic flavor, strange flavors and oiliness). 

The panelists evaluated each attribute using an unstructured 15-cm analog scale ranging from “none” (0) to “very” (15). Sensory analysis was also performed immediately after sample preparation. For texture and flavor attributes, the sensory analysis was also performed 30 min after sample preparation. The samples were kept at 74 °C in a dish warmer during those 30 min. In each session, three samples were delivered in coded plastic plates in random order. Every panelist was provided with spring water to cleanse the palate between samples. The results from the analog scale were converted to numerical values (0 to 15).

### 2.10. Statistical Analysis

The measurements obtained for rheological characteristics, batter pick-up, moisture content, oil content and color determination were analyzed using one-way ANOVA followed by Tukey’s post-hoc test, and values of *p* < 0.05 were considered significant. For the analysis of sensory and texture characteristics, differences between means were analyzed using a two-way analysis of variance (ANOVA) followed by Tukey’s pair-wise comparison test, with the same threshold for statistical significance. Statistical analyses were performed with GraphPad Prism 5 (GraphPad Software, Inc., San Diego, CA, USA). The values obtained for the rheological analysis were graphically fit to rheological models by optimizing R2.

## 3. Results and Discussion

### 3.1. Batter Rheology Characterization 

In the three batter formulations studied, viscosity decreased with increasing shear rate, revealing a shear-thinning pattern typical of batters [4]. Data from the flow curves were well fitted to the Herschel–Bulkley model (R^2^ = 0.98–0.99). The yield stress (σo), consistency index (Keq) and flow index (n) for the different batters are given in Table 2.

Batter viscosity is a function of several variables including ingredients (especially protein and starch), particle size, temperature, solid-to-water ratio and the amount of free water present [19,20]. In this study, EB samples had a significantly higher consistency index and lower yield stress and flow index than SB and MB samples. MB samples had the lowest consistency index, which could be explained by the partial substitution of wheat flour by maltodextrin. Modification of starches, as occurs in maltodextrin, also influences the apparent viscosity. Maltodextrin is a short-chain carbohydrate obtained by the enzymatic hydrolysis of starches. It has been reported that viscosity decreases as the particle size of the batter decreases [21]. Our results support this conclusion, as the partial substitution of flour by maltodextrin decreased the viscosity; similar findings were previously reported by Baixauli et al. (2003). However, the higher consistency index observed in EB samples was discordant with the results reported by other authors. Xue and Ngadi (2006) observed a 63% viscosity decrease in batters containing 50% wheat flour and 50% corn flour compared to the batter containing 100% wheat flour. This finding was attributed to the ability of wheat gluten to absorb water, resulting in decreased free water in the batter system, whereas corn flour proteins do not absorb water as easily and thus dilute the strengthening influence of wheat flour gluten. The lower amount of water in EB samples resulting from partial ingredient substitution with ethanol could explain this difference. None of the previous studies in which ethanol was included in the batter formulation analyzed the resulting rheological characteristics. 

### 3.2. Batter Pick-up

The measurement of batter pick-up is important because this parameter is closely associated with the appearance, crispiness and thickness of the external crust in deep-fried foods. The analysis of batter pick-up for the three formulations is summarized in Table 3. SB samples had significantly higher batter pick-up whereas the maltodextrin batter resulted in the lowest pick-up values. In general, pick-up values range from 30% to 50% [22]. Our results were consistent with this range except for the MB samples, which had a significantly lower average pick-up value of 17.9%. These results are in accordance with those reported in previous studies. It has been widely demonstrated that, as viscosity increases, more batter remains on the sample, resulting in higher pick-up values [1,20,23,24]. 

We also found that zucchini slice thickness affected batter pick-up. The thinnest slices retained significantly more batter than the thickest ones (9.3% difference).

### 3.3. Color and Visual Appearance 

**Figure 1:**
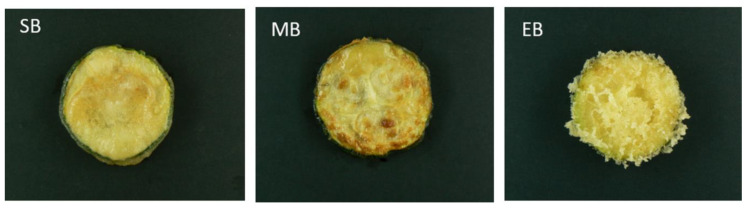
External appearance of different tempura batter zucchini samples after frying during 260 s at 170 °C. SB: standard batter; MB: maltodextrin batter; EB: ethanol batter.

The external appearance after the frying process of each sample covered by different batter formulation is shown in Figure 1. SB samples showed a smooth and uniform surface over the product. MB products presented a less uniform and thinner coating on its surface while EB samples resulted in a rough surface, with higher volume and a higher number of air gaps. This appearance could be caused by the presence of a leaving agent and the faster evaporation and high diffusivity of the ethanol in the batter [14]. In addition, Altunakar et al. (2004) found that the addition of corn starch to a batter resulted in a higher surface porosity.

The L*, a*, b* color parameters of the fried samples processed with the three different batter formulations are shown in Table 3. As expected, longer frying times resulted in lower L* values, indicating that the samples were darker. Moreover, the addition of maltodextrin to the batter formulation significantly influenced the lightness parameter, as MB samples had a darker surface than SB and EB samples. This could be explained by the greater presence of chain ends with reducing capacity in maltodextrin-containing flours compared with other flours, which results in a higher frequency of non-enzymatic browning reactions [25]. 

Batter formulation also affected the red component of color more than the yellow component, and MB samples had higher a* values. This behavior was previously observed by other authors in different fried food matrix-like battered squid rings [14,26]. 

### 3.4. Texture Analysis

The results of the texture analysis are shown in Table 4. EB samples had significantly lower maximum force values, and higher count peaks and workforce than the other batter formulations immediately after frying. MB and SB samples only differed with respect to maximum force immediately after frying, with MB samples having the highest values.

All studied parameters decreased after 30 min in the three batter formulations, except for work force in the EB samples. However, the workforce and count peaks were different across the three batter formulations. The workforce and count peaks hardly changed in the MB and EB samples, whereas they drastically decreased in SB samples. This finding could be related to the moisture content, as moisture is a common plasticizer that promotes the mobility of polymers. Thus, the moisture concentration affects the mechanics of the protein and starch matrix and the resulting texture characteristics [27].

Our results are concordant with those reported by Carvalho and Ruiz (2018), who found that ethanol-containing batters had lower maximum force values and a higher number of fracture events than batters containing only water and that these characteristics did not change over time. 

A high number of fracture events is associated with a crisp product and high force values indicate more resistance to cutting, i.e., a less fragile covering [28,29]. It was also observed that a higher ratio of ruptures on the surface is associated with a higher loss of moisture and higher oil uptake [30], as we found in our study. EB samples showed a higher amount of count peaks and oil-uptake.

### 3.5. Moisture and Oil Content

The moisture and oil content of fried samples processed with the three different batter formulations are shown in Table 3. 

There was no significant difference in moisture content between samples prepared with SB or MB formulations; in contrast, the samples with added ethanol (EB) had significantly lower moisture content. These results are concordant with those reported by Carvalho and Ruiz–Carrascal (2018), who found that there was 31.8% less moisture in samples prepared with ethanol compared to those prepared with only water. In this study, the differences in moisture content between the samples were not as marked, probably because our batter formulations were different from those used in the Carvalho study.

Oil uptake is influenced by a variety of factors such as oil quality, frying temperature, frying time, pre-treatments, and food composition [5,31], as well as the size, shape and initial moisture content of the surface of the food [3]. The mechanism of oil uptake involves heat transfer, evaporation of surface water and the migration of oil droplets into the resulting gaps. Thus, when the water losses during frying are reduced, the oil uptake also decreases [5]. The results of the present study are concordant with those of previous studies: SB and MB samples, which had higher moisture content, also had lower oil content, whereas EB samples had less moisture but more than double the oil content (oil content 17.8%, 15.8% and 36.9% for SB, MB and EB respectively). Furthermore, the higher porosity and size of gaps on the surface of EB, caused by the ethanol, baking powder and corn starch in this batter, facilitates the inside oil retention. Similar results were observed by [32] and [14] in fried chicken nuggets covered by a batter containing corn flour and in fried battered squid rings with ethanol in the tempura dough, respectively. In contrast, a higher proportion of wheat flour in batter results in higher moisture content and the development of a strong thermoplastic film that acts as a barrier against water evaporation, leading to less oil uptake [33].

Frying time has also been observed to affect oil uptake. The final oil and moisture content values were significantly higher and lower, 54% and 43% respectively, after 360 s of frying compared with those with 180 s of frying. This could be explained by the higher rate of dehydration with an increase in frying time. Similar results were observed by Rahimi and Ngadi (2014). We also found that the thickest slices had the lowest oil uptake, 17.8%, which could be due to the higher moisture content of the sample core. 

### 3.6. Sensory Analysis

**Figure 2:**
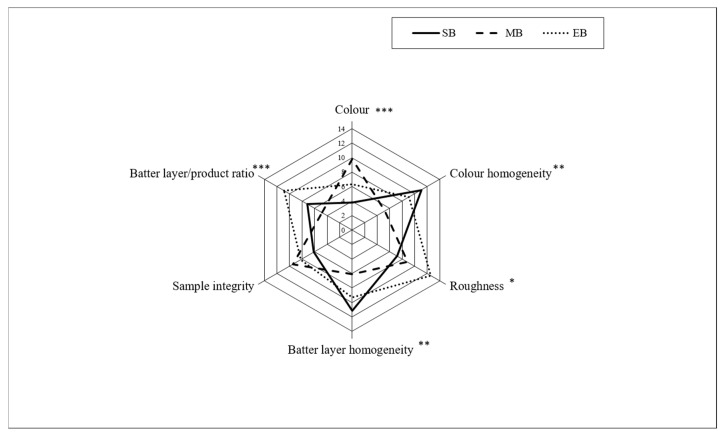
Results of the sensory analysis of the external characteristics of fried samples (*n* = 30). SB: standard batter; MB: maltodextrin batter; EB: ethanol batter. Color SEM = 1.02; Color homogeneity SEM = 1.01; Roughness SEM = 0.92; Batter layer homogeneity SEM = 0.89; Sample integrity SEM = 0.72; Batter layer/product ratio SEM = 0.90. Significant differences between samples are expressed by * *p* < 0,05; ** *p* < 0.01; *** *p* < 0.001.

The results of the sensory analysis of external aspects of fried zucchini battered with the three different formulations immediately after frying are shown in Figure 2.

Batter formulation significantly affected the external appearance of the sample. The panelists found MB samples to be significantly darker in color than the other samples, which was concordant with the results of the instrumental analysis. The MB samples received the lowest scores for the batter layer/product ratio, which is consistent with our finding that MB samples had the lowest pick-up values.

The scores for roughness, which represent the magnitude and density of surface asperities [34], were highest for the EB samples. This could be explained by the effect of the leavening agent as well as the higher ratio of ruptures on the surface due to the faster and more explosive evaporation of ethanol.

The texture and flavor attributes as measured in the sensory analysis immediately and 30 min after frying are shown in Table 5. The crispiness is probably the most expected characteristic texture in fried products. The results of the sensory analysis showed that samples coated with the EB formulation were the crispiest immediately after frying. This result was consistent with the results of the instrumental analysis, as EB samples also had the highest number of count peaks. Carvalho ans Ruiz–Carrascal (2018) also observed higher crispiness in batters with ethanol in their sensory analysis. SB and EB samples had a lower number of count peaks and a loss of crispiness 30 min after frying, but the sensory analysis was not able to discriminate the degree of loss as accurately as the instrumental analysis. 

Regarding flavor attributes, there was no significant difference in juiciness between the different batter formulations but EB samples received higher scores for oiliness. This perception could be due to the significantly higher oil uptake of the EB formulation. Similar attributes of ethanol-based tempura samples were observed by Carvalho and Ruiz–Carrascal (2018). In our study, the addition of ethanol to the dough formulation and the partial substitution of wheat flour with cornflour gave the product a distinct flavor, which was retained over time. As a consequence, the characteristic flavor intensity scores of the EB samples were lower, especially immediately after frying. 

## 4. Conclusions

The thickness of slices, frying time and tempura batter formulation affected the final oil content, texture and sensory characteristics of coated and fried vegetables such as zucchini. Thinner slices and longer frying times resulted in higher oil uptake. The partial substitution of wheat flour with maltodextrin in the batter is an effective alternative which can be used to obtain a more intense golden–brown color in the final product, with a lower batter layer–product ratio according to the sensory analyses, lower pick-up, so that less expenditure batter, and slightly less final oil content.

Conversely, the inclusion of ethanol, a leavening agent and cornflour in the tempura batter resulted in the crispiest coating, with desirable higher surface roughness. However, the greater moisture loss and porosity of the crust also resulted in higher oil absorption.

Varying tempura batter ingredients according to the desired purpose could be of interest to industrial manufacturers and gastronomic centers. The inclusion of maltodextrin can improve important organoleptic characteristics in fried products, such as color, reducing at the same time the pick-up of the batter, while the addition of ethanol and leavening agents can result in improved and longer-lasting crispiness of the crust.

## Figures and Tables

**Table 1:** Comparison of the coating batter formulations.

Coating Batters			
Batter Ingredients	Standard Batter (SB)	Maltodextrin Batter (MB)	Ethanol Batter (EB)
Wheat Flour (g)	80	60	50
Corn Flour (g)	-	-	30
Maltodextrin (g)	-	20	-
Mineral Water at 15 °C (mL)	125	125	75
Salt (g)	1	1	1
Sugar (g)	1	1	1
Ethanol (g)	-	-	50
Baking Powder (g)	-	-	5

**Table 2:** Rheological characteristics of the different batter formulations fitted to the Herschel–Bulkley model.

Coating Batter	Yield Stress (s_o_)	Consistency Index (K_eq_)	Flow Index (n)	R^2^
SB	219.30 ± 25.10 ^a^	0.40 ± 0.37 ^a^	1.42 ± 0.29 ^a^	0.991
MB	133.70 ± 13.30 ^b^	0.11 ± 0.06 ^b^	1.41 ± 0.09 ^a^	0.981
EB	103.60 ± 10.47 ^c^	5.68 ± 1.82 ^c^	0.84 ± 0.09 ^b^	0.997

SB: standard batter; MB: maltodextrin batter; EB: ethanol batter. Mean values ± standard deviations. Letters in each column indicate significant differences (*p* < 0.05) (*n* = 24).

**Table 3:** Differences in the pick-up, moisture, dry matter, final oil content and color of samples prepared with different coating batters and conditions.

Coating Batter	Time Frying (s)	Slice Thickness (mm)	Pick-up (%)	Moisture (%)	Oil Content (%)	Color		
						L*	a*	b*
SB	180	4.25	29.8 ± 1.2 ^a^	64.0 ± 1.2 ^a^	15.3 ± 0.3 ^a^	78.8 ± 1.7 ^a^	-0.5 ± 0.3 ^a^	26.3 ± 2.1 ^a^
SB	260	4.25	31.3 ± 0.9 ^a^	58.2 ± 0.9 ^a^	17.8 ± 1,4 ^a^	75.9 ± 1.8 ^a^	4.3 ± 1.4 ^b^	34.0 ± 1.9 ^b^
SB	360	4.25	32.2 ± 1.0 ^a^	48.2 ± 4.3^b^	23.6 ± 2.4 ^b^	71.1 ± 3.4 ^b^	6.7 ± 1.3 ^c^	31.3 ± 3.7 ^b^
SB	260	2.25	40.7 ± 3.2 ^b^	39.2 ± 3.6 ^c^	27.3 ± 2.4 ^b^	76.0 ± 1,9 ^a^	3.8 ± 0.8 ^b,d^	31.4 ± 2.2 ^b^
SB	260	3.00	40.2 ± 1.8 ^b^	45.7 ± 2.3^b,c^	23.7 ± 1.5 ^b^	76.0 ± 1.8 ^a^	3.2 ± 0.6 ^b,d^	33.6 ± 1.5 ^b^
SB	260	4.25	31.3 ± 0.9 ^a^	58.2 ± 0.9 ^a^	17.8 ± 1.4 ^a^	75.9 ± 1.8 ^a^	4.3 ± 1.4 ^b^	34.0 ± 1.9 ^b^
SB	260	4.25	31.3 ± 0.9 ^a^	58.2 ± 0.9 ^a^	17.8 ± 1.4 ^a^	75.9 ± 1.8 ^a^	4.3 ± 1.4 ^b^	34.0 ± 1.9 ^b^
MB	260	4.25	17.9 ± 0.7 ^c^	59.5 ± 2.2 ^a^	15.8 ± 2.2 ^a^	70.4 ± 3.7 ^b^	6.0 ± 1.5 ^c^	32.1 ± 1.8 ^b^
EB	260	4.25	28.5 ± 1.3 ^d^	41.2 ± 3.7 ^b,c^	36.9 ± 0.3 ^c^	77.6 ± 1.3 ^a^	2.7 ± 0.8 ^d^	31.7 ± 1.9 ^b^

SB: standard batter; MB: maltodextrin batter; EB: ethanol batter. Mean values ± standard deviations. Letters in each column indicate significant differences (*p* < 0.05). Sample sizes were as follows: pick-up measurement, *n* = 30; measurement of moisture, dry matter and oil content, *n* = 9; color analysis, *n* = 12.

**Table 4:** Results of the texture analysis after instrumental analysis of samples with different coating batters.

Coating Batter				
	Time (min)	SB	MB	EB
Force (Kg)	0	1.6 ± 0.2 ^a,^*	2.3 ± 0.3 ^b,^*	1.1 ± 0.2 ^c,^*
	30	0.2 ± 0.0 ^a,^†	1.8 ± 0.2 ^b,^†	1.4 ± 0.2 ^c,^†
Area (Kg·s)	0	6.9 ± 1.3 ^a,^*	5.7 ± 0.7 ^a^ *	8.0 ± 0.9 ^b,^*
	30	1.0 ± 0.4 ^a,^†	4.1 ± 0.7 ^b,^†	7.3 ± 0.9 ^c,^*
Count Peaks	0	29.0 ± 4.2 ^a,^*	26.0 ± 6.7 ^a,^*	38.6 ± 8.3 ^b,^*
	30	2.2 ± 1.6 ^a,^†	16.7 ± 3.5 ^b,^*	26.8 ± 5.5 ^c,^†

SB: standard batter; MB: maltodextrin batter; EB: ethanol batter. Mean values ± standard deviations. Different symbols (*,†) in each column indicate significant differences between time points within each batter type (*n* = 15). Different lowercase letters (a,b,c) in each row indicate significant differences between batter types at the same timepoint (*n* = 15).

**Table 5:** Texture and flavor characteristics of fried samples as assessed by sensory analysis immediately and 30 min after sample preparation.

	SB	MB	EB	SEM	*p_bat_*	*p _bat × time_*
Hardness						
0 min	5.52 ^a,^*	5.33 ^a,^*	7.86 ^b,^*	0.45	0.018	0.1024
30 min	5.66 ^a,^*	4.17 ^a,^*	6.13 ^a,^†	0.35	0.077	
*p _time_*	0.531	0.295	0.020			
Crispness						
0 min	9.72 ^a,^*	7.34 ^b,^*	11.21 ^c,^*	0.60	0.0002	0.3637
30 min	8.69 ^a,^†	7.96 ^a,^*	9.49 ^a,^†	0.30	0.218	
*p _time_*	0.0018	0.895	0.030			
Juiciness						
0 min	9.92 ^a,^*	8.98 ^a,^*	8.84 ^a,^*	0.40	0.548	0.8943
30 min	9.92 ^a,^*	8.43 ^a,^*	8.32 ^a,^*	0.45	0.307	
*p _time_*	0.740	0.522	0.691			
Characteristic flavor intensity						
0 min	8.24 ^a,^*	9.76 ^a,^*	5.59 ^b,^*	0.75	0.036	0.222
30 min	8.05 ^a,^*	9.25 ^a,^*	8.01 ^a,^*	0.55	0.321	
*p _time_*	0.268	0.747	0.355			
Strange flavour						
0 min	0.57 ^a,^*	1.00 ^a,^*	5.29 ^b,^*	0.75	<0.0001	0.7183
30 min	0.44 ^a,^*	0.95 ^a,^*	4.03 ^b,^*	0.78	0.002	
*p _time_*	0.640	0.863	0.379			
Oiliness						
0 min	7.84 ^a,^*	7.92 ^a,^*	10.51 ^b,^*	0.76	0.003	0.2201
30 min	8.48 ^a,^*	8.98 ^a,^*	8.92 ^a,^*	0.15	0.807	
*p _time_*	0.565	0.252	0.088			

SB: standard batter; MB: maltodextrin batter; EB: ethanol batter. Data show mean values and standard error of the mean (SEM). Different symbols (*,†) in each column indicate significant differences between time points within each batter type (*n* = 30). Different lowercase letters (a,b,c) in each row indicate significant differences between batter types at the same timepoint (*n* = 30).
